# Analysis of Correlation between Climate Change and Human Health Based on a Machine Learning Approach

**DOI:** 10.3390/healthcare9010086

**Published:** 2021-01-17

**Authors:** Vito Alberto Pizzulli, Vito Telesca, Gabriela Covatariu

**Affiliations:** 1 School of Engineering, University of Basilicata Macchia Romana, Viale dell’Ateneo Lucano 10, 85100 Potenza, Italy; vitoalberto.pizzulli@gmail.com; 2 Faculty of Civil Engineering and Building Services, Gheorghe Asachi Technical University of Iasi, Prof. Dimitrie Mangeron Blvd. 65, 700259 Iași, Romania; gabriela.covatariu@tuiasi.ro

**Keywords:** environmental conditions, mortality cases, morbidity cases, neural networks, artificial intelligence, forecast

## Abstract

Climate change increasingly affects every aspect of human life. Recent studies report a close correlation with human health and it is estimated that global death rates will increase by 73 per 100,000 by 2100 due to changes in temperature. In this context, the present work aims to study the correlation between climate change and human health, on a global scale, using artificial intelligence techniques. Starting from previous studies on a smaller scale, that represent climate change and which at the same time can be linked to human health, four factors were chosen. Four causes of mortality, strongly correlated with the environment and climatic variability, were subsequently selected. Various analyses were carried out, using neural networks and machine learning to find a correlation between mortality due to certain diseases and the leading causes of climate change. Our findings suggest that anthropogenic climate change is strongly correlated with human health; some diseases are mainly related to risk factors while others require a more significant number of variables to derive a correlation. In addition, a forecast of victims related to climate change was formulated. The predicted scenario confirms that a prevalently increasing trend in climate change factors corresponds to an increase in victims.

## 1. Introduction

Climate change is an increasingly recurring theme in recent years, from science to politics, attracting the attention of more and more people. Although there is scientific proof of climate change, there are numerous opinions and different approaches to combatting it. The European Commission’s 2020 climate and energy package aims to reduce greenhouse gas emissions (compared to 1990 levels) and energy needs from renewable sources, as well as improve energy efficiency all by 20%.

The Fourth Assessment Report [1] included sectoral and regional analyses of published literature related to climate change impacts. The Report considered the weight of evidence supporting its conclusions and attributed relative levels of confidence.

CMIP5 is one of the most commonly used models in climate change [2]. NASA and other entities that study climate variations over time use it and have added other methods like ModelE2 for the atmospheric model [3]. The ModelE2 atmospheric general circulation model (AGCM) has a resolution of 2.5° longitude by 2° latitude and 40 vertical layers. Hydrostatic approximation is introduced using pressure as a vertical coordinate, with terrain-following sigma coordinates in the 23 layers below 150 mb. The thickness of the layer above the pressure level is horizontally uniform to the top of the model at 0.1 mb. With six layers below 825 mb and the tropopause, with nine layers between 251 and 43 mb, the vertical resolution is improved near the lower boundary.

For the CMIP5 experiments, three variants of ModelE2, characterized by their treatment of atmospheric composition and aerosol indirect effects (AIE), were used. Aerosols indirectly disrupt radiative fluxes by acting as nuclei of cloud condensation (CCN), altering the clouds’ brightness and lifespan. Specifically, aerosols reduce cloud droplet size by distributing condensed water over a more significant number of nucleation sites (the ‘‘Twomey’’ or first AIE) [4], and these smaller droplets increase the number of collisions required for the droplets to grow to precipitable size (the ‘‘Albrecht’’ or second AIE) [5].

An example is the calculation of instantaneous forcing which represents the difference between CMIP3 and CMIP5, demonstrating the new method’s better accuracy (CMIP5).

Some of the many diseases that are present globally have increased in diffusion and have exhibited a worsening of symptoms, and this may be due to climage change. According to the World Health Organization, 23% of deaths in the world are attributable to environmental causes, thus about 4 million people [1]. Deaths caused by the environment can be traced back to some pathologies that have been favored by climate change. The IPCC set out in Climate Change 2007: Working Group II: Impacts, Adaptation and Vulnerability, the best model used until 2007 to estimate and predict Health effect and Climate scenario [1].

Climate change currently contributes to the global burden of disease and premature deaths (with very high confidence). Human beings are exposed to climate change by changing weather patterns [6] (temperature, precipitation, sea-level rise, and more frequent extreme events) and indirectly through water, air, and food quality and changes in ecosystems, agriculture, industry, and settlements and the economy. At this early stage, the effects are small but are projected to increase in all countries and regions progressively.

The increase in the concentration of CO_2_ in the air and the consequent increase in temperature are causing considerable damage to the environment, human life and human health. The influences of weather and climate on human health are significant and varied. They range from explicit threats of extreme temperatures and severe storms to connections that may seem less obvious [7]. For example, weather and climate affect the survival, distribution, and behavior of insects that carry diseases, particularly in arid zones. Climate and weather can also affect water and food quality in particular areas, with implications for human health [8]. Furthermore, the effects of global climate change on mental health and well-being are powerful, constituting a considerable risk for humans [9].

A useful approach to understanding how climate change affects health is to consider specific exposure pathways and how they can lead to human disease [1]. The definition of exposure pathways is adapted from its use in chemical risk assessment and explains the key routes by which health is influenced by climate change in this context. Exposure pathways could affect humans in different ways, depending on the condition and timing at the location Climate exposures are subject to single or multiple changes and geographical position, factors which potentially affect human risk.

Threats to climate change can also build up over time, leading to longer-term adaptation and health improvements, and they depend on a complex collection of susceptibility factors such as whether or not a person is exposed to a health hazard or is suffering from disease or other adverse health effects from that exposure [6]. Vulnerability is the tendency or predisposition to be adversely affected by climate-related health effects and encompasses three elements: exposure, sensitivity, or susceptibility to harm, and the capacity to adapt or cope.

Models to analyze human health are also difficult to formulate as they are often inaccurate. Linking human health with weather is quite laborious because there are too many variables to consider, for example people’s behavior, genetic disease, attention paid to medical check-ups, and regional variations [10].

There are various models to find a correlation between human health and climate change (i.e., statistical, regression, artificial intelligence), but it depends on what is analyzed and how much data is available. One of the best studies on health is WHO04, the only current quantitative study of the impacts of global warming on diarrhea [11]. Using empirical studies from Fiji and Peru [12], the WHO04 investigation inferred that warming by 1 °C was associated with a 5% increase in diarrhea and noted that this was probably a conservative estimate.

A broad range of uncertainty (0–10% per 1 °C) was added to the association between diarrhea and temperature, but temperature estimates from a single climate model were used. As mentioned above, it is standard practice to use multi-model assemblies (a collection of results from multiple models) when evaluating the spatial and temporal aspects of climate predictions and forecasts due to the considerable amount of intermodel difference concerning regional predictions [13,14].

Starting from temperature data and the CMIP3 model, the authors calculated a scenario for the next few years, hypothesizing that temperature is increasing worldwide. Since temperature is one of the causes of increases in diarrhea cases, they evaluated the consequent increase in diarrhea cases [12].

Additionally, the models that allow prediction of a future scenario, using the data and previously mentioned correlations, are relevant [14]. Studies highlight an estimated rise in deaths due to warming in the summer months (hot season, April–September), a predicted decline in deaths due to warming in the winter months (cold season, October–March), and an expected net shift in deaths for the U.S. cities studied [15,16]. These observations equate estimated deaths for future reporting to 1990 outcomes while maintaining population at 2010 levels and without any methodological modification for possible future adaptation. Thus, temperature–death associations found for the last decade of the available evidence (1997–2006) are expected to remain unchanged in 21st century forecasts [17,18].

Based on these assumptions, the result is an increasing health benefit in terms of reduced deaths during the cold season (October–March) over the 21st century due to warming temperatures, while deaths during the hot season (April–September) increase [19]. Overall, in the hot season, increased deaths from warming outweigh a decrease in deaths during the cold season, resulting in a net rise in deaths attributed to climate change attributable to temperature over time.

These studies have almost all focused on examining the correlation between a disease and some aspects of climate change, referring to a specific area, usually a city or a nation [10]. In this work a framework based on Artificial Intelligence techniques has been developed to analyze the correlation between climate variability and main mortality diseases on global scale, and to predict a future scenarios by the tested best correlation models, using as input a climate change forecast developed by NASA [2].

## 2. Materials and Methods

The data used in this research naturally concern climate change and human health. First, it is necessary to delimit the study area, on which the search for data of both types will be carried out. Most previous studies were carried out on a regional basis, or if worldwide facts were considered different mathematical models were applied for each selected region.

In the present case, analysis is conceived on a global scale. Although there are substantial climatic and health differences among the countries analyzed, the use of a single mathematical model for each analyzed typology increases its accuracy. Because the input data is obtained through the same mathematical methodology, there is no need to make adjustments or corrections, thus reducing errors significantly. Having unified data on a global scale means there is only one source, therefore data is in a single format, making it more convenient to analyze.

The aim is to find a correlation between climate change and human health on a global scale, trying to understand which diseases are most affected by climate factors and finally, to make a forecast on their mortality, to understand what actions should be taken to mitigate this risk.

**Figure 1:**
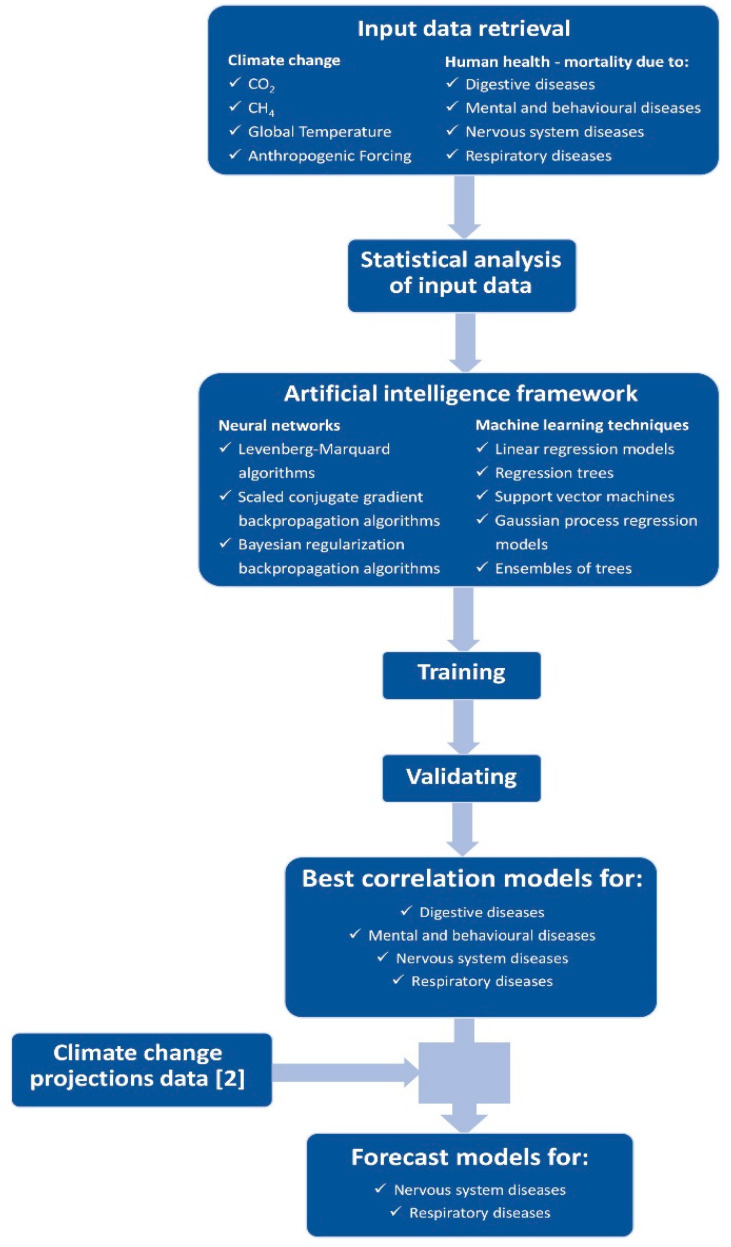
Methodology used in this work.

Figure 1 outlines the methodology used in this work, from input data retrieval to results and forecast models.

As mentioned, only one source was chosen for climate data and another for human health data: NASA (NASA database, 2020) [20] and WHO (WHO database, 2020) [21], respectively. As these are the most reliable sources available, veracity and reliability of the data are maximized.

Climate change data were selected based on previous scientific research that identifies four main drivers of climate change with the most significant impact on human health: temperature, CO_2_, CH_4_ and anthropogenic forcing.

The NASA GIIS database provides a variety of downloadable data about climate; in the present case, the data was taken from previous research [2] that studied the forced model of climate change worldwide.

The WHO periodically publishes an assessment of the impact of climate change and recent studies have expanded the research to all components related to climate change, especially the impact on human health. Mortality data were selected from the research carried out by the World Health Organization as they are the most reliable on a global scale and, above all, many similar studies use the same data, thus a comparison between them and this work is correct and accurate. The WHO mortality database allows downloading of victim data by applying different filters. For this study all countries in the world, all deaths by causes of disease, and all years available from the database were chosen.

First, the total number of worldwide deaths every year, minus the deaths caused by accidents, was analyzed to support the research. Data from 1979 to 2016 were examined but errors emerged due to missing data from some especially poor countries that did not provide this information.

From the number of global total deaths, four causes were chosen which are considered related to climate change and have attracted more attention over the years [22,23]: mental and behavioral disorders, respiratory disease, nervous system disease, and digestive disease. This data also came from the WHO and spanned from 1979 to 2016 with some missing data from the beginning and end of the period.

An analysis period from 1980 to 2015, during which all the necessary data were available, was selected.

Table 1 reports a pre-analysis of the data, with calculation of the principal statistics, useful for featuring scale.

Matlab tools (Matlab, 2020) [24,25], Neural Network toolbox, and Deep Learning Toolbox were used for the analysis, all three artificial intelligence techniques based on the same principle. In this case, we used two different types of AI, the simplest neural networks, as they are composed of a single level which allowed us to understand if there was a correlation between climate change and human health. Subsequently, using a multi-level neural network, such as Machine Learning and Deep Learning, we tried to improve the performance of the obtained correlation and understand if the studies carried out with the neural networks were correct.

The neural networks analysis was conducted in order to simulate the correlation between input (CO_2_, CH_4_, temperature and anthropogenic forcing) and output data (four causes of death [22,23]). Once the network is trained to be used on different input values, the correspondent output values simulated by the network can be computed.

The Regression Learner app was used to explore the data, to select features, train models, and assess results, and it reports the validated model’s performance. Diagnostic metrics, such as model precision, and charts, such as response plot or residuals plot, represent the validated model outcomes. The app can automatically train one or more regression models, compare the effects of validation, and choose the best model that fits the regression issue. The exported model can be used to make predictions on new data.

## 3. Results

### 3.1. Analysis with Neural Networks

The neural networks simulate the correlation between the environment conditions (temperature, CO_2_, CH_4_, anthropogenic forcing) and the mortality and morbidity (the number of deaths without external causes, number of cases for several diseases like mental and behavioural disorder, respiratory, nervous system and digestive).

In a first step, neural networks were used to verify whether or not there was a correlation between the pathologies and the climatic variables chosen. Neural networks have limits, which can sometimes be overcome by implementing a different methodology, and in this case machine learning techniques were applied.

Several configurations were tested for the neural network. Starting from a hidden layer and 10 neurons, progressively increasing the number of layers of one and the neurons by 10, analysis testing was carried out to determine which configuration gives the best performance value.

Finally, a feedforward network with one hidden layer, and 4-10-5 configuration (four neurons on input layer, 10 neurons on hidden layer and five neurons on output layer) was chosen. Several algorithms were used to train the network, including Levenberg-Marquard (*trainlm*), Scaled conjugate gradient backpropagation (*trainscg*) and Bayesian regularization backpropagation (*trainbr*) algorithm (Figure 1). The data were normalized (due to different ranges for data) to train the network.

The data were randomly divided into three sets: 70% for training, 15% for validation, and 15% for testing.

**Figure 2:**
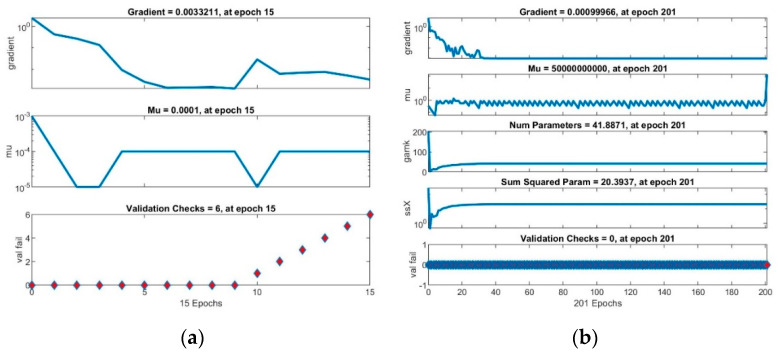
Neural networks performance: (**a**) with Levenberg-Marquardt algorithm; (**b**) with Bayesian regularization backpropagation algorithm.

**Figure 3:**
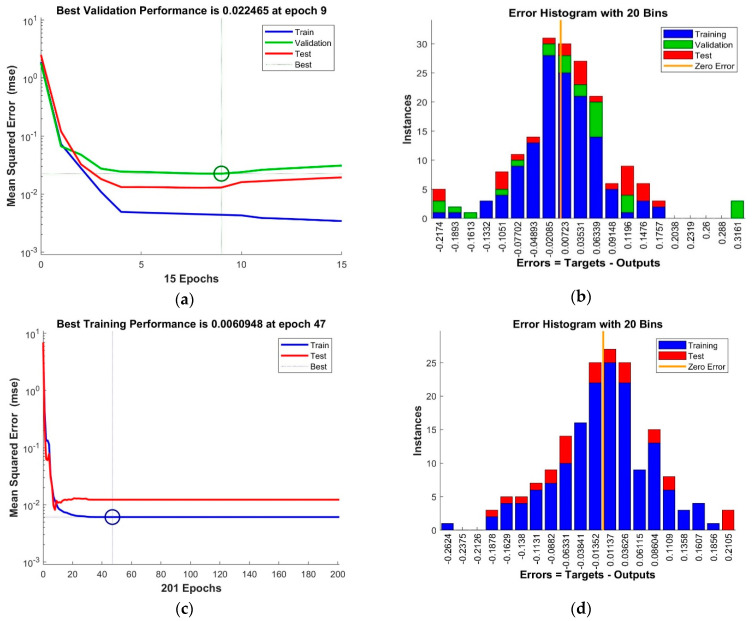
Neural networks performance: (**a**,**b**) with Levenberg-Marquard algorithm; (**c**,**d**) with Bayesian regularization backpropagation algorithm.

The training of the neural networks was concluded when the performance measure stopped improving, the maximum mu (adaptation parameter) was reached (Figure 2b and Figure 3a) or validation error MSE (Mean Squared Error) was at minimum (Figure 2a and Figure 3c).

The resulting error histograms (Figure 3b,d) show small errors, with apparent normal distribution and central tendency close to 0.

**Figure 4:**
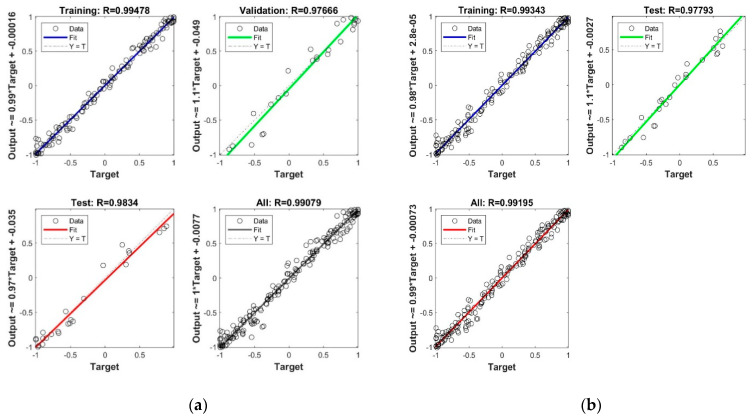
Neural networks performance: (**a**) with Levenberg-Marquard algorithm; (**b**) with Bayesian regularization backpropagation algorithm.

The correlation coefficient R^2^ (Figure 4) between output and target values in the neural networks training process has outstanding values, close to 1.

Overall, neural networks analysis shows that a good correlation (Table 2) between these variables can be found. The forecasted data for CO_2_, CH_4_, and temperature can be used, with the help of the neural network already educated and the machine learning app, to determine the prognosis of the number of deaths or other numbers of illness cases and create global and human evolution scenarios.

### 3.2. Analysis with Regression Learner (Machine Learning App in MATLAB)

The Regression Learner app from MATLAB was used to train regression models to predict data. The first analysis was made using data sets with four input values and one output value for each studied variable.

**Figure 5:**
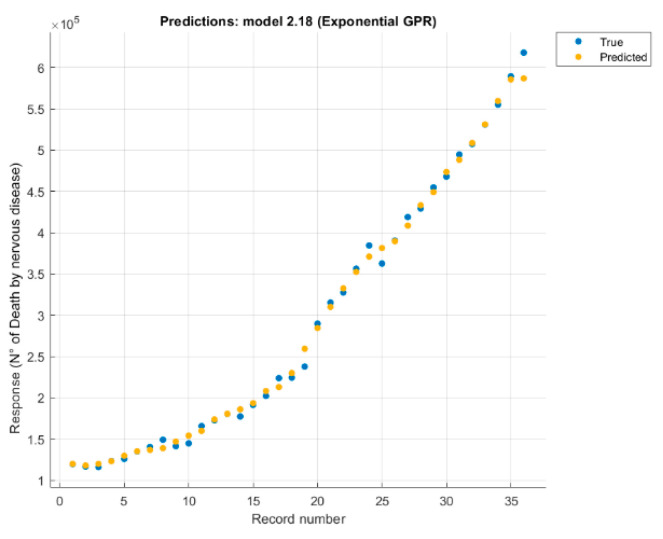
Response plot for nervous system disease deaths, predicted with three features, using Exponential Gaussian Process.

**Figure 6:**
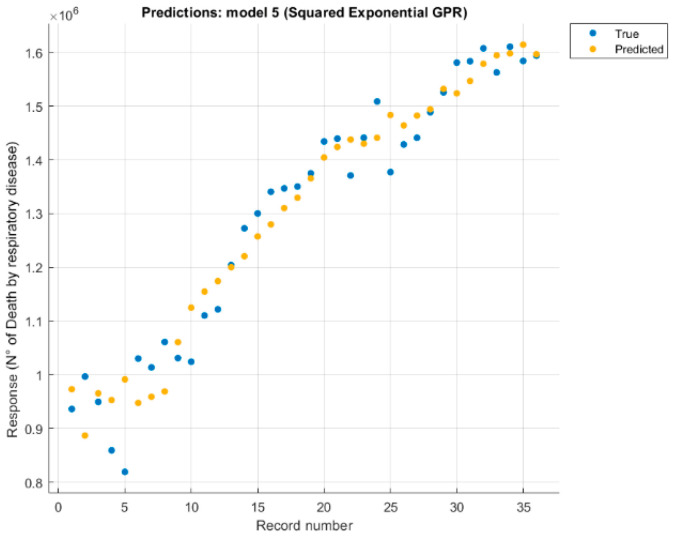
Response plot for respiratory disease deaths, predicted with three features, using Squared Exponential Gaussian Process.

It was studied separately, each variable (number of deaths without external causes just disease, digestive disease, mental and behavioural disorder, nervous system disease, respiratory disease) correlated with four causes (CO_2_, CH_4_, temperature, and the anthropogenic forcing) values. Each case changed the configuration and retrained the model, eliminating causes one by one, until the best option was found (Figure 5 and Figure 6).

**Figure 7:**
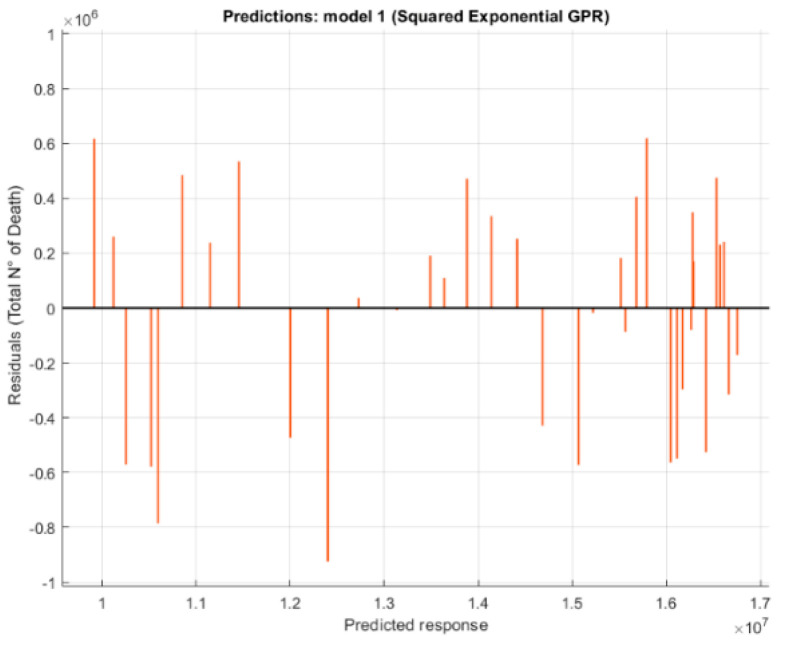
Residual plot for number of deaths predicted with two features, using Exponential Gaussian Process.

The residuals plot (Figure 7) was used to check model performance. The residuals plot displays the difference between the predicted and true responses.

Analysis of the data reveals good correlation (Table 3) between climate change and the four diseases studied, however, due to the amount of data, the correlation between climate change and digestive diseases is dictated by an increasing trend of input and output data, therefore it is not to be trusted.

### 3.3. Assumption About Future Trends

The impacts of developmental, climatic and environmental scenarios on population health are essential for healthcare planning processes. Furthermore, future trends in health are relevant to climate change because the health of populations is an important element of adaptive capacity. In this way, the prediction function in Matlab was used, applying the same previously obtained model but entering new data in the input referring to future years.

The future forecasts data come from NASA and refer to a scientific publication [2] predicting the variation in concentration of some gases in the air which are particularly related to climate change. The input data used to obtain the models in the previous analysis are reported until 2016. The forecasts use data from 2017 and beyond, depending on the type of forecast requested. The data used from 2017 to 2019 are real data, derived from satellite scans, processed as previously described as an annual average over the entire globe. For data after 2019, reference is made to the scientific publication mentioned above [2].

The prediction about the four variables (CO_2_, CH_4_, temperature, and anthropogenic forcing) is based on the CMIP5 model, which uses satellite data relating to the variables and produces a prediction model based on the hypothesis that human activity changes remain those in force. The forecasts are made on the basis of the main regulations in force regarding the reduction of polluting emissions and an increase in the use of energy from renewable sources.

Almost all future trends predict an increase in climate change variables. Therefore it is reasonable to expect an increase in future scenarios as well.

The new input data, which also contains the future trends, must be entered in Matlab with the same procedure used previously, importing from excel only the columns used. They must also be arranged in the same order as the model analysis.

After exporting a model to Regression Learner’s workspace, a trainedModel structure was used to make predictions using new data. The structure contains a model object and a function for prediction. The structure enables predictions for models that include principal component analysis (PCA): yfit = trainedModel.predictFcn(T), where trainedModel is the name of the exported variable, and T is the data with the same format and data type as the training data used (table or matrix).

From those five models derived using the regression learner app in machine learning, only two will simulate a future trend. The models with the least error (RMSE), are those related to the victims due to nervous system disease. The performance of some pathologies is almost perfect, due to the almost growing trend of input and output data. The results show that two pathologies have almost the same characteristics (mental and behavioral and nervous system disease), therefore a single future scenario will be created. Therefore, a similar trend is expected in the near future.

**Figure 8:**
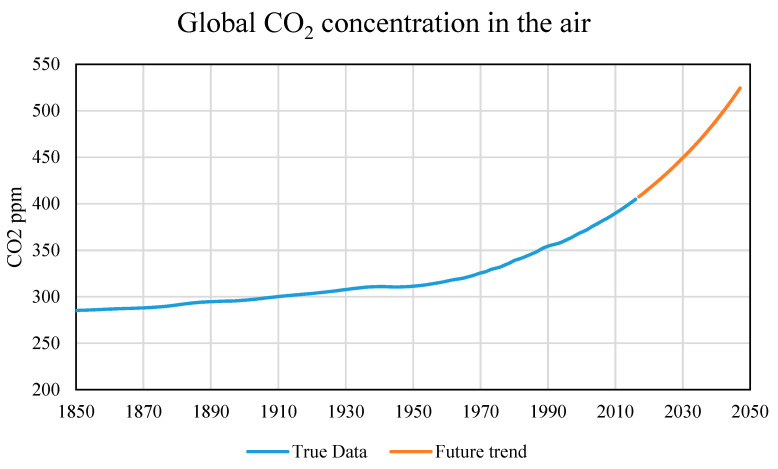
Global CO_2_ concentration in the air: true data [2] and future trend.

The forecast was made until the year 2500. However, being the result of a mathematical model, such a distant forecast is not reliable, therefore predictions will be made for 10-year intervals, using the predictive data for all user input data (Figure 8) in the following figures relating to the four previously used climate variables.

**Figure 9:**
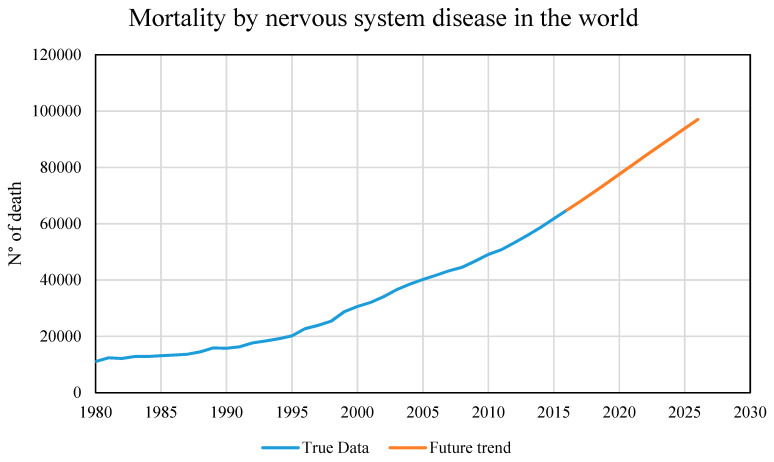
Mortality due to nervous system disease: true data and future trend.

In Figure 9, the scenario has only been evaluated for the next 10 years, although it is possible to do it up to 500 years data, but making a forecast for 500 years is neither reliable and affected by high uncertainty. The intention is to show a minimal correlation, however with more detailed data and more significant variables, a more accurate forecast could be obtained.

The predicted number victims from nervous system disease are quite significant: in 35 years the increase is almost 5000 more deaths per year, and in only 10 years the predicted increase is 3000 deaths. The significant increase in casualties due to climate change each year will lead to a greater increase in the coming years than in the past.

**Figure 10:**
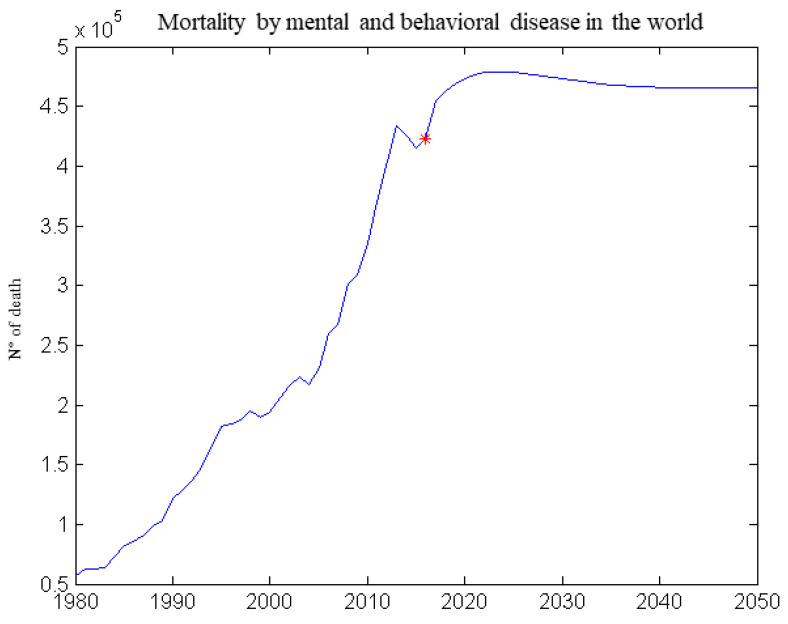
Mortality due to mental and behavioral disease (forecast created using neural networks).

The best model obtained was used to produce a forecast, despite the correlation being due to an almost increasing trend of the input and output curves. Therefore it was decided to create a forecast using neural networks, to try to overcome some problems obtained from regression through machine learning. Using the forecast data for Temperature, CO_2_, CH_4_, and anthropogenic forces we simulated with the help of the neural network already educated and we obtained forecasts for all studied variables, but in Figure 10 the forecast for the nervous system can be seen.

As can be seen in Figure 10, also the forecast obtained from the neural networks shows us an increasing trend, but by increasing the number of years taken into consideration, a decrease in deaths is observed. The presence of a decrease in deaths may mean that neural networks have produced slightly better results for this type of model.

**Figure 11:**
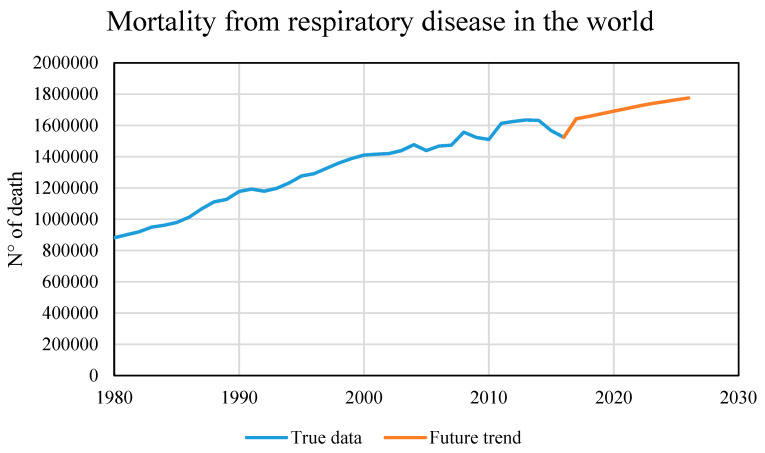
Mortality from respiratory disease (true data and future trend).

Similarly, a future 10-year scenario was created based on the model referring to the victims of respiratory diseases. Figure 11 shows a possible future scenario, 10 years as before, but with a notable difference: the increase in casualties is not so remarkable but is almost linear with the curve of real data. Not having used a linear method for the predictive model, the almost linear trend suggests that the temperature variable’s fluctuating trend, which is fundamental in this model, can also influence the future trend.

As mentioned above, this model is the most relevant and the one that best suits the incoming climate data, although it has a lower R^2^ index than the other trained models. Deaths due to respiratory diseases are strongly related to climate change and in particular to temperature changes, to temperature fluctuations such as heatwaves or frost and especially to the constant annual increase in the Earth’s average temperature.

## 4. Discussion

The results obtained from neural networks allow us to state that there is a correlation between climate change and human health on a global scale, in accordance with previous studies [26]. A more in-depth analysis using machine learning techniques confirmed the correlation again, returning more detailed information on the environmental risk factors most closely linked to the diseases studied.

Mental and behavioral disease and nervous system disease are closely related to each other given the nature of the pathology, and therefore it is possible to consider them together. Mental pathologies are strongly correlated to climate change; they have the lowest relative error of all the pathologies analyzed.

Mathematically, the correlation between climate change and respiratory diseases is less reliable than those mentioned above, despite relative error being 6%. However, the close correlation between temperature and respiratory diseases, regardless of the other causes of climate change, shows an excellent fit between the input and output trends, confirming the correlation between climate change and digestive diseases.

Study of the correlation between climate change and human health using artificial intelligence has produced new results. Two different types of artificial intelligence were used to analyze the correlation better.

Our study using neural networks produced excellent results, furthering the understanding of which input variables has the most significant influence in the analysis. The machine learning technique confirmed the previously obtained correlation, improving the reliability of three of the four pathologies studied.

The resulting forecasts do not have a very high reliability, but comparing the scenarios deduced with those of other scientific articles it is possible to say that they are very similar and that the error is mainly due to the limited quantity of data and the nearly infinite number of variables necessary to describe such a complex phenomenon. The use of global data has simplified the calculation but, of course, analyzing only certain countries and only certain types of mortality which are predominant in the selected countries, the results could be better. The difficulty to obtain data on human health is a sizeable obstacle to studying correlations between it and climate change.

## 5. Conclusions

The present study examined correlations between climate change and human health on a global scale, using available multi-annual monitoring data, through analysis with artificial intelligence techniques.

Previously identified correlations [1] expressing the link between climate change and human health on a continental scale, suggest that mortality related to some specific diseases has been valid for two types of diseases (respiratory disease and nervous system disease) [27].

Studying the correlations using artificial intelligence has produced new results. Two different types of artificial intelligence were used in order to analyze the best correlation.

Our study using neural networks has produced excellent results, furthering the understanding of which input variables has the most significant influence in the analysis. The machine learning technique confirmed the previously obtained correlation, improving the reliability of three of the four pathologies studied.

The analyses carried out with artificial intelligence techniques have yielded better results than purely statistical ones. In particular, the analysis with machine learning techniques generated the model with the fewest absolute error.

No correlation was found between climate change and victims of digestive problems. This mortality variable should be analyzed using different cause variables linked to water and food quality and their availability rather than the leading causes of climate change.

The correlation between climate change and victims of mental and nervous system disorders produced unreliable results, dictated by the curve’s nearly linear and easy to predict trend, suggesting a more detailed analysis is need to confirm its validity.

The correlation found between climate change and victims of respiratory disease is the most valid, with temperature playing a fundamental role in this correlation, confirming previous studies [27].

A possible future scenario was forecast for two of the diseases analyzed, showing that there will be an increase in the number of victims of respiratory and mental diseases due to climate change in the next ten years [28].

The overall analysis carried out in this study produced less detailed results, compared to previous studies, but showed that despite the large area analyzed it is still possible to find a correlation between climate change and human health.

In conclusion, there is undoubtedly a correlation between climate change and human health, especially between temperature change and deaths due to respiratory diseases. However, having more data available and focusing analysis on a narrower geographical area would produce better results.

## Figures and Tables

**Table 1:** Statistical indicators for input data (1980–2015) from NASA and WHO (victim numbers are based on yearly global deaths).

	Temperature Anomaly (°C)	CO_2_ (ppm)	CH_4_ (ppb)	Anthropogenic Forcing (W m^−2^)	Victims Without External Causes	Mental and Behavioural Disorder	Respiratory Diseases	Nervous System Diseases	Digestive Diseases
mean	0.034	318.58	1.17	0.941	13,446,963.9	192,014.5	1,247,399.7	281,894.2	640,789.2
stdev	0.341	32.18	0.35	0.926	3,677,068.5	115,776.1	324,588.9	162,370.5	172,157.3
min	−0.482	285.20	0.79	0.000	1,393,261.0	28,739.0	105,959.0	41,322.0	69,373.0
25%	−0.211	295.00	0.86	0.245	11,490,490.8	93,066.5	1,030,557.0	142,181.3	515,161.0
50%	−0.071	309.50	1.05	0.625	14,483,355.5	185,460.5	1,348,770.0	224,029.0	644,557.0
75%	0.209	332.20	1.48	1.417	16,037,828.0	252,202.0	1,476,771.5	411,736.8	787,771.5
max	1.015	410.40	1.88	3.421	17,006,389.0	433,349.0	1,610,627.0	618,126.0	850,382.0

**Table 2:** The performance of the chosen neural networks (HL = Hidden Layer).

Training Algorithm	Number of Neurons on HL	Number of Iterations	NN Perform MSE	Gradient	Mu	R2
*trainlm*	10	15	0.0034	0.0033	0.0001	0.9908
	20	9	0.0027	0.0035	0.0001	0.9922
	25	10	0.0006	0.0024	0.0025	0.9899
*trainscg*	10	34	2.83	2.8700		0.9885
	20	14	0.0161	0.0481		0.9550
	25	58	0.0053	0.0124		0.9897
*trainbr*	10	158	0.0054	0.0009	5 × 10^10^	0.9991
	20	201	0.0061	0.00096	5 × 10^10^	0.9919
	25	346	0.0078	0.0011	5 × 10^10^	0.9912

**Table 3:** Results of the analysis with the Regression Learning model.

Analyzed Variable	Regression Model	R^2^	Best Correlation
Total number of Deaths without accidents	Gaussian Squared Exponential Process	0.96	CO_2_, CH_4_
Digestive disease	Gaussian Exponential Process	0.98	CO_2_, CH_4_, temperature
Mental and behavioral disease	Gaussian Exponential Process	0.99	CO_2_, CH_4_, anthropogenic forcing
Nervous system disease	Gaussian Exponential Process	1.00	CO_2_, CH_4_, anthropogenic forcing
Respiratory disease	Gaussian Squared Exponential Process	0.94	CO_2_, CH_4_, temperature

## Data Availability

Climate data (NASA database): Available online: https://data.giss.nasa.gov/modelE/efficacy/. Mortality data (WHO database): Available online: https://apps.who.int/healthinfo/statistics/mortality/whodpms/.
